# The Intensity of Heat Exchange between Rock and Flowing Gas in Terms of Gas-Geodynamic Phenomena

**DOI:** 10.3390/e23050556

**Published:** 2021-04-29

**Authors:** Katarzyna Kozieł, Juliusz Topolnicki, Norbert Skoczylas

**Affiliations:** The Strata Mechanics Research Institute, Polish Academy of Sciences, 30-059 Kraków, Poland; koziel@imgpan.pl (K.K.); juliusztopolnicki@gmail.com (J.T.)

**Keywords:** rock–gas heat transfer, gas and dolomite outburst, energy balance, energy stored in gas, determining the outburst risk

## Abstract

Gas-induced geodynamic phenomena can occur during underground mining operations if the porous structure of the rock is filled with gas at high pressure. In such cases, the original compact rock structure disintegrates into grains of small dimensions, which are then transported along the mine working space. Such geodynamic events, particularly outbursts of gas and rock, pose a danger both to the life of miners and to the functioning of the mine infrastructure. These incidents are rare in copper ore mining, but they have recently begun to occur, and have not yet been fully investigated. To ensure the safety of mining operations, it is necessary to determine parameters of the rock–gas system for which the energy of the gas will be smaller than the work required to disintegrate and transport the rock. Such a comparison is referred to as an energy balance and serves as a starting point for all engineering analyses. During mining operations, the equilibrium of the rock–gas system is disturbed, and the rapid destruction of the rock is initiated together with sudden decompression of the gas contained in its porous structure. The disintegrated rock is then transported along the mine working space in a stream of released gas. Estimation of the energy of the gas requires investigation of the type of thermodynamic transformation involved in the process. In this case, adiabatic transformation would mean that the gas, cooled in the course of decompression, remains at a temperature significantly lower than that of the surrounding rocks throughout the process. However, if we assume that the transformation is isothermal, then the cooled gas will heat up to the original temperature of the rock in a very short time (<1 s). Because the quantity of energy in the case of isothermal transformation is almost three times as high as in the adiabatic case, obtaining the correct energy balance for gas-induced geodynamic phenomena requires detailed analysis of this question. For this purpose, a unique experimental study was carried out to determine the time required for heat exchange in conditions of very rapid flows of gas around rock grains of different sizes. Numerical simulations reproducing the experiments were also designed. The results of the experiment and the simulation were in good agreement, indicating a very fast rate of heat exchange. Taking account of the parameters of the experiment, the thermodynamic transformation may be considered to be close to isothermal.

## 1. Introduction

Gas-induced geodynamic phenomena are a frequent problem in mining [1,2]. The most serious are outbursts of rocks and gas, defined as dynamic destruction of the original rock structure along with transportation of the disintegrated material into the interior of the working space. In a typical outburst, several hundred tons of rock are disintegrated to grains less than a centimeter in size and transported tens of meters along the mine working space. Outbursts pose a significant danger to miners and to the mine infrastructure. Outbursts may occur in porous rocks if the porous space and crevices of the rock are filled with gas at high pressure [3,4,5]. Many authors analyze gas-geodynamic phenomena in an attempt to estimate what energy is stored in the gas contained in the pore structure. However, it is difficult to indicate any work that examines the operation of the gas during its sudden decompression, experimentally or by modeling. This results in frequent presumptions as to the nature of the thermodynamic transformation. The novelty described in the article is the analysis of the nature of the transformation taking place during a sudden gas decompression [6].

This problem is most often associated with coal mining [7,8,9,10]. However, beside coal mines, the risk of rock and gas outbursts also exists in potassium and salt mines [11,12] and in sandstone formations above coal strata [13].

In the past decade, in Polish copper mining regions, the presence of gas in the pores of copper-bearing rocks has been reported with increasing frequency, particularly in carbonate rocks [14]. The first serious outburst in a copper ore mine took place on 6 September 2009 [15] at the O/ZG Rudna mine. Another incident classified as a rock and gas outburst occurred in 2018 at the O/ZG Polkowice-Sieroszowice mine. The sizes of the caverns formed by the respective outbursts were 250 and 219 m^3^. Reports were made on both incidents, and investigations showed the main cause of the outbursts to be the presence of gas in dolomite [16,17].

## 2. Energy Balance

Analysis of the nature of gas-induced geodynamic phenomena requires a qualitative and quantitative determination of the effect of individual parameters on the probability of occurrence and course of events. A fundamental problem in such cases is determination of the energy balance, which enables a proportional comparison of the factors affecting accumulation of energy in the rock–gas system and the work that must be done during phases of the event [18]. The main additive component in the energy balance for a gas and rock outburst is the potential energy of the gas released in the course of sudden decompression. During the outburst, the rock is disintegrated, and the products are transported into the mine working space [19]. Both destruction and transportation are fueled mainly by the energy of decompression of the gas originally located in the pores of the disintegrated rock. The quantity of released energy depends strongly on the course of the process of decompression. The energy balance may be expressed by the Equation (1).

(1) $$E_{G}=W_{R}+W_{T}$$

where:

$E_{G}$ is the energy of the gas during decompression from the rock [J];
$W_{R}$ is the work required to disintegrate the rock [J];
$W_{T}$ is the work required to transport the rock into the working space [J].

### 2.1. Work of Disintegration

The work done by the gas in disintegrating the rock can be experimentally investigated by subjecting samples to tensile loads [20]. If we additionally apply elements of Rittinger’s theory [21], the work of disintegration of a unit volume of rock may be obtained as proportional to the surface area of the disintegrated rock fragments, and the coefficient of proportionality obtained by dividing the work required to break a sample in a Brazilian test by the fracture surface area of the sample. The work of disintegration is given by the Equation (2).

(2) $$W_{R}=\frac{W_{b}}{p_{p}}\cdot p_{r1}$$

where:

$W_{b}$ is the work done in breaking a sample in the Brazilian test [J/m^3^];
$p_{p}$ is the fracture surface area of the sample [m^2^];
$p_{r1}$ is the surface area corresponding to a unit volume of rock (1 m^3^) after its disintegration into grains of the size classes identified in examination of the outburst material [m^2^/m^3^].

Tests carried out on dolomites to determine the tensile strength of the rock have shown that the energy required to disintegrate 1 m^3^ of rock is approximately 0.38 MJ.

### 2.2. Work of Transporting the Rock Along the Working Space

The remainder of the energy of the gas is absorbed in transporting the disintegrated rock into the mine working space. As a result of the outburst of gas and rock at the O/ZG Rudna mine, rock was deposited in the working space over a length of approximately 70 m [19,22,23]. The work done in transporting 1 m^3^ of rock one half of the distance covered by the outburst material was estimated using the Equation (3).

(3) $$W_{T}=\rho \cdot g\cdot f_{d}\cdot s$$

where:

ρ is the rock density [kg/m^3^];
g is acceleration due to gravity [m/s^2^];
$f_{d}$ is the coefficient of kinetic friction [−];
s is the distance travelled by the outburst material [m].

The energy needed to be supplied by the gas to transport the rock is approximately 0.70 MJ/m^3^. Thus, from the work of disintegration and the work of transportation, it is possible to calculate the total energy of the gas required for the geodynamic event to take place:

(4) $$E_{G}=0.38+0.70=1.08\;MJ/m^{3}$$

## 3. Energy of the Gas

To determine the parameters at which a rock and gas outburst is possible (porosity and gas pressure in the rock) it is necessary to establish the nature of the thermodynamic transformation taking place during decompression of the gas from the rock pores. The importance of the porosity is that it determines the amount of gas that can be released from a given volume of rock, e.g., if the volume of a rock is V and the porosity is ε%, the volume of the gas at atmospheric pressure is εV. Considering the seriousness of the danger posed by outbursts, this a very important issue for miner safety. The problem also applies to copper ore mines, with copper metal being a key raw material in modern economies [24].

### 3.1. Isothermal Transformation

In thermodynamics, four basic types of transformation of gas are distinguished, defined by a set of thermodynamic variables: pressure (*p*), temperature (*T*), and volume of the gas (*V*) [25]. During a gas and rock outburst, the gas that initially filled the volume of the porous space in the rock undergoes decompression, which lowers its pressure and increases its volume [26]. For this reason, we reject isochoric and isobaric transformation. If we assume that the process is isothermal, this means that, in practice, the exchange of heat between the gas and the surrounding rock is sufficiently effective that the temperature of the gas remains unchanged or changes only to a marginal degree. It is known from the ideal gas state Equation (5) that the volume of gas is inversely proportional to its pressure, as described by the Boyle–Mariotte law:

(5) $$V=\frac{const}{p}$$

which allows us to write:

(6) $$p_{0}V_{0}=p_{1}V_{1}$$

where:

$p_{0}$ is the gas pressure in the pore in the rock bed;
$p_{1}$ is the pressure in the mine working space (0.11 MPa);
$V_{0}$ is the volume of gas at pressure $p_{0}$;
$V_{1}$ is the volume of gas after transformation.

The main component of the gas mixture in the pores of copper-bearing rocks is nitrogen, which may be treated as an ideal gas. The energy of the gas during decompression may be written as

(7) $$W_{G}=\int_{V_{0}}^{V_{1}}p\left(V\right)dV$$

This equation may be expanded to obtain a formula for the energy in isothermal transformation:

(8) $$W_{Gi}=nRT\int_{V_{0}}^{V_{1}}\frac{1}{V}dV=nRT{\left[ln\left|V\right|\right]}_{V_{0}}^{V_{1}}=nRT\;ln\left(\frac{V_{1}}{V_{0}}\right)\;=nRT\;ln\left(\frac{p_{0}}{p_{1}}\right)$$

(9) $$W_{Gi}=p_{0}V_{0}ln\left(\frac{p_{0}}{p_{1}}\right)$$

**Figure 1:**
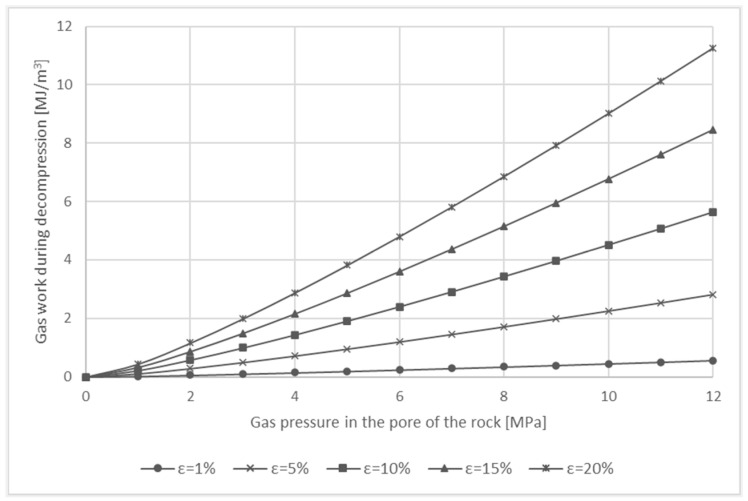
Isothermal gas work released during decompression of gas from the porous structure of the rock, depending on porosity.

Figure 1 shows a theoretical graph of the work of the gas depending on the pressure and the porosity of the rock in the case of isothermal transformation. The gas in the rock is located in the pores of the rock. The gas volume depends on the porosity of the rock. The pressure of the gas contained in the porous structure may reach 12 MPa, and the porosity of the rock ranges from 1% to 20%. 

### 3.2. Adiabatic Transformation

Gas-induced geodynamic phenomena may also potentially be describable as adiabatic transformations. A condition for an adiabatic process is the absence of heat exchange with the surroundings. For that condition to be fulfilled, either the process must take place in a well-insulated container, which is not possible in the case of mines, or it must occur extremely rapidly, with negligibly small heat exchange. In adiabatic transformation, there is no exchange of heat:

(10) δQ=0

The energy in the case of adiabatic transformation may be expressed by the formula

(11) dU=Q−W

(12) dU=−W

(13) $$W_{a}=nc_{v}\Delta T$$

where the specific heat is given by

(14) $$c_{v}=\frac{R}{k-1}$$

and hence

(15) $$W_{a}=\frac{nR}{k-1}\left(T_{0}-T_{1}\right)=\frac{nRT_{0}}{k-1}\left(1-\frac{T_{1}}{T_{0}}\right)$$

Then, by Poisson’s law

(16) $$p_{0}V_{0}^{k}=p_{1}V_{1}^{k}$$

we obtain

(17) $$\frac{T_{1}}{T_{0}}={\left(\frac{p_{1}}{p_{0}}\right)}^{\frac{k-1}{k}}$$

Thus, the work done in the adiabatic process is given by the formula

(18) $$W_{a}=\frac{p_{0}v_{0}}{\kappa -1}\left(1-{\left(\frac{p_{1}}{p_{0}}\right)}^{\frac{\kappa -1}{\kappa }}\right)$$

where:

κ = 1.4 is the adiabatic index.

**Figure 2:**
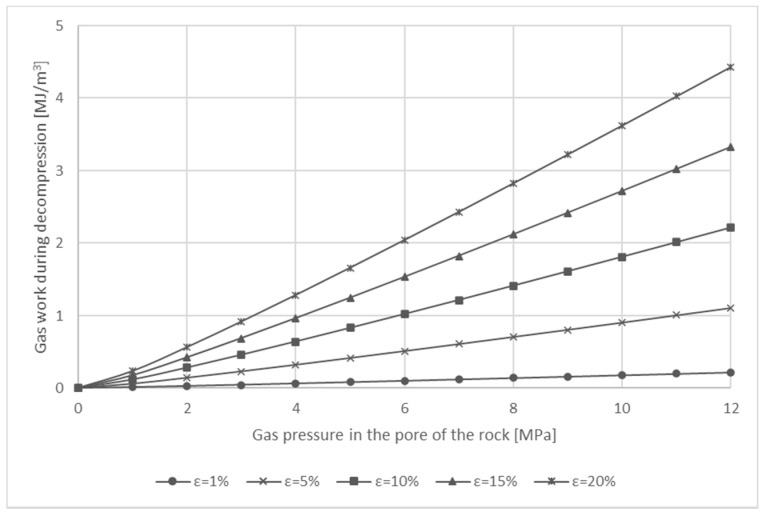
Adiabatic gas work released during decompression of gas from the porous structure of the rock, depending on porosity.

Figure 2 shows theoretical values of the adiabatic energy of the gas in a geodynamic event. 

Comparing the two processes, there a significant difference is seen between the values of energy obtained for the same parameters. For example, taking typical values for the porosity of dolomites (ε = 5%) [27,28,29] and for the pressure of gas in the porous structure (*p* = 6.5 MPa) [30,31,32], the value of the energy obtained for an isothermal process is approximately 1.33 MJ/m^3^, while for an adiabatic transformation it is approximately 0.56 MJ/m^3^. For the same gas pressure, assuming a higher porosity of 20%, the energy value is almost 3 MJ/m^3^ higher for isothermal than for adiabatic transformation. This means that it is extremely important to establish the nature of the transformation that occurs during a rock and gas outburst. This knowledge can be used to establish limiting parameters of the rock-gas system for safe copper ore mining operations. 

## 4. Experimental Methodology

### 4.1. Specific Heat

To determine the nature of the transformation taking place during gas-induced geodynamic events, it is necessary to estimate the ability of the gas to receive heat from fragments of disintegrated rock. The first step is to determine the specific heat of the studied rock [33]. An experiment was carried out as follows: a known mass of dolomite was heated to the temperature *t_d_* and was then transferred to a calorimeter partially filled with water (the temperature of the water was equal to that of the calorimeter). The heat received by the calorimeter and the water contained in it is given by

(19) $$Q_{P}=m_{w}\cdot c_{ww}\cdot \left(t_{u}-t\right)+m_{k}\cdot c_{wk}\cdot \left(t_{u}-t\right)$$

where:

$m_{k}$ is the mass of the calorimeter [kg];
$m_{w}$ is the mass of water [kg];
$c_{ww}$ is the specific heat of water [J/kg*K];
$c_{wk}$ is the specific heat of the calorimeter [J/kg*K];
t is the temperature of the water and of the calorimeter [K];
$t_{u}$ is the stabilised temperature [K].

The heat given up by the dolomite is

(20) $$Q_{O}=m_{d}\cdot c_{wd}\cdot \left(t_{d}-t_{u}\right)$$

where:

$m_{d}$ is the mass of heated dolomite [kg];
$c_{wd}$ is the specific heat of dolomite [J/kg*K];
$t_{d}$ is the temperature to which the dolomite was heated [K].

The specific heat of the dolomite $c_{wd}$ is given by the Equation (22)

(21) $$m_{d}\cdot c_{wd}\cdot \left(t_{d}-t_{u}\right)=m_{w}\cdot c_{ww}\cdot \left(t_{u}-t\right)+m_{k}\cdot c_{wk}\cdot \left(t_{u}-t\right)$$

(22) $$c_{wd}=\frac{\left(m_{w}\cdot c_{ww}+m_{k}\cdot c_{wk}\right)\cdot \left(t_{u}-t\right)}{m_{d}\cdot \left(t_{d}-t_{u}\right)}$$

### 4.2. Heat Exchange during Gas Flow through a Bed

**Figure 3:**
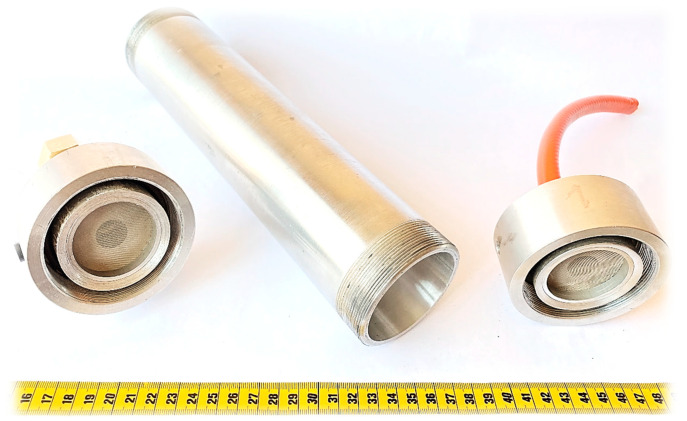
Aluminum pipe used in the experiment as a container for disintegrated rock.

**Figure 4:**
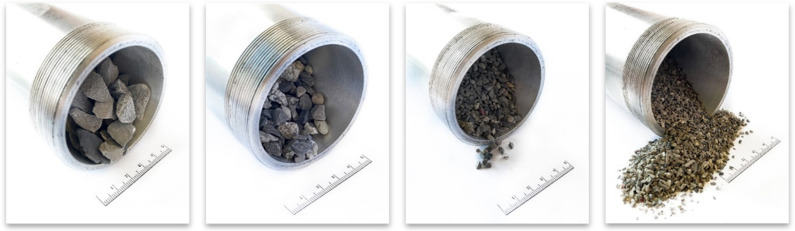
Rock material prepared for the experiment, in four different grain size classes.

An experiment was carried out with gas flowing through a dolomite bed. Grains of rock were prepared in four size classes: 1–2, 2–4, 4–8, and 8–20 mm. In the experiment, dynamically decompressed gas flowed through a bed of dolomite grains, and the temperature was measured at three points: the two ends of the bed and its midpoint. The principal aim of the experiment was to measure the change in temperature between the points of inflow and outflow so as to determine how fast the cooled flowing gas could be heated by the dolomite. The experiment was conducted in an aluminum pipe (Figure 3) containing rock material with grain sizes corresponding to those of the rock from the outburst at the O/ZG Rudna mine (Figure 4).

**Figure 5:**
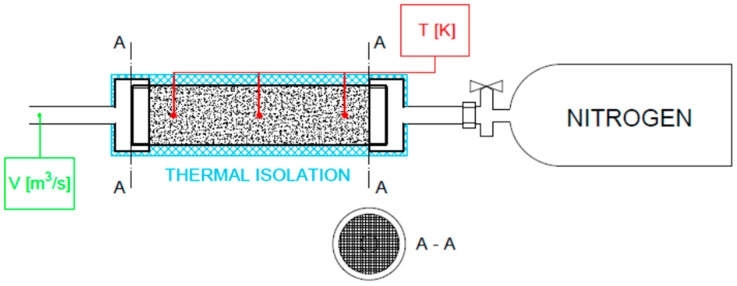
The measuring stand.

Temperature sensors (unscreened type K thermocouples) with low time constants were placed at three points in the pipe—the entrance, the midpoint, and the exit—at equal intervals of 100 mm. The pipe had a total length of 250 mm and a diameter of 60 mm. Steel sieves were placed at both ends of the pipe to hold the rock grains in place. One end of the pipe was connected directly to a bottle containing nitrogen (180 bar, 50 L). When the bottle was fully opened (without a regulator) it produced a very high flow rate of gas, which was cooled by several tens of degrees during decompression. A schematic diagram of the measuring stand is given in Figure 5. During the experiment, the pipe was wrapped in a thermal insulating jacket. The volume of the gas stream was measured at the other end of the pipe.

The aim of the experiment was to determine whether the rock was capable of heating some portion of the highly cooled gas to a temperature close to that of the rock.

### 4.3. Model Study

**Figure 6:**
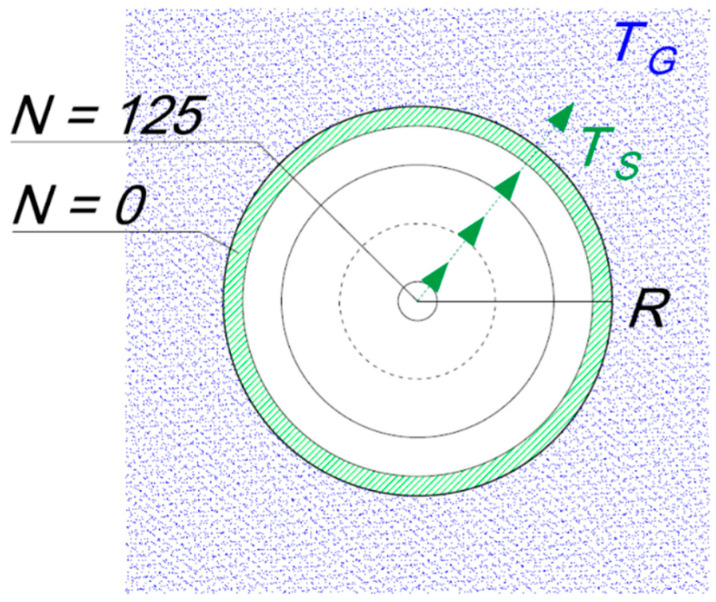
Rock-gas heat exchange.

**Figure 7:**
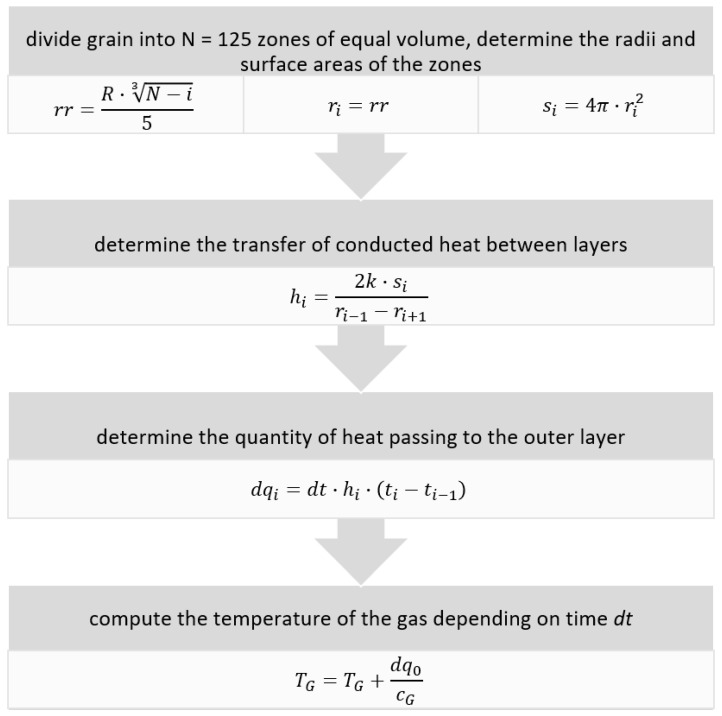
Algorithm for the program implementing the grain–gas heat exchange model.

**Figure 8:**
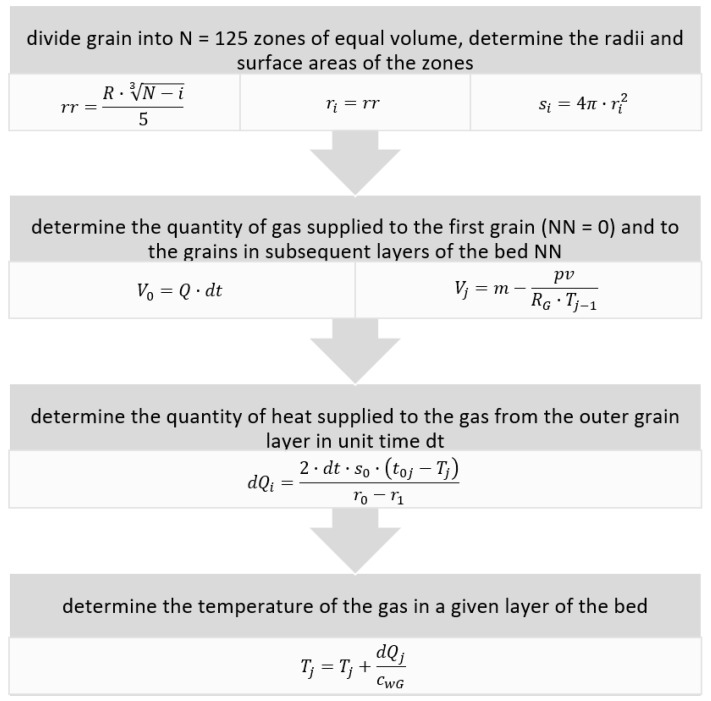
Algorithm for the program implementing the heat exchange model.

The rate of heat exchange is dependent on the total surface area of the rock grains, their temperature, the temperature of the gas, the specific heat of the rock, and the heat capacity of the gas. In the initial stage, the dominant form of heat exchange is the flow of heat from the surface of the grains to the gas. To determine the nature of the transformation of the gas, a series of simulations and experiments was carried out (Figures 7 and 8). To model the process of heat exchange in the rock–gas system a spherical grain shape was assumed, this being the least favorable for heat exchange. Simulations were performed for a grain size of 10 mm as that size class accounts for the greatest proportion of the outburst material. A spherical grain of radius *R* was divided into *N* layers of equal volume. The model experiment assumes that the rock is initially at a uniform room temperature of 300 K. The grain is surrounded by gas at a temperature of around 80 K. The temperature of the decompressed gas surrounding the grain was obtained from Equation (17), taking the initial temperature to be 300 K, the pore pressure to be 100 bar, and the final pressure to be equal to atmospheric pressure. Layer 0 is in contact with the gas and with layer 1 of the dolomite. Heat is exchanged between neighboring layers; heat streams flow from the inner layers (*N*), warming up the outer layer, which is being cooled by the gas. Figure 6 shows a schematic diagram of the heat exchange process in the rock-gas system.

The program implementing the heat exchange model uses the following algorithm (Figure 7):

where:

N is the number of layers in the grain;
i is the index of a layer;
R is the grain radius [5 × 10^−3^ m];
$r_{i}$ is the radius of the *i*-th layer;
$s_{i}$ is the surface area of the *i*-th layer;
$h_{i}$ is the transfer of conducted heat;
k is the thermal conductivity of the rock;
$dq_{i}$ is the quantity of heat flowing to the outer layer from the *i*-th layer;
dt is the time step [1 × 10^−9^ s];
$t_{i}$ is the temperature of the *i*-th layer;
$T_{G}$ is the temperature of the gas depending on the number of time steps *dt*;
$c_{G}$ is the heat capacity of the gas.

The quantity of released gas depends on the porosity of the rock and the pressure of the gas contained in its porous structure. In gas-geodynamic phenomena, the gas is released from the pore structure of the rock, and to illustrate the amount of gas, the relationship between the volume of gas and the volume of the rock was used. If the volume of the rock is V and the porosity is, e.g., 10%, then the volume of gas at atmospheric pressure will be 0.1V. If the gas in the rock pores is under higher pressure, then the gas volume will be expressed as the product of rock volume, porosity, and pressure. Table 1 gives the assumed volumes of gas, expressed in terms of the grain volume (V), for different values of the parameters of the rock–gas system.

This model is an attempt to reproduce the phenomenon occurring during a rock and gas outburst in mine conditions, where the quantity of emitted gas is strongly dependent on its pressure and on the porosity of the rock and, in such a perspective, is proportional to the quantity of grains [13]. Based on given parameters of the rock–gas system, the program models the changing temperatures of the rock and gas. To extend the model to represent a bed of grains, which would enable verification of the experimental phase of the study, the program’s algorithm was modified, although it was still based on a spherical grain model. The modified algorithm modeling the conducted experiment is given below (Figure 8).

where:

$V_{0}$ is the portion of gas supplied to layer 0 of the bed;
$V_{j}$ is the portion of gas passing to layer NN_j_;
Q is the rate of inflow of the gas;
m is the quantity of gas surrounding the sample together with the supplied gas;
p is the pore pressure;
v is the grain volume;
$R_{G}$ is the universal gas constant;
$T_{j-1}$ is the temperature of the gas in the *j*-th layer.

**Figure 9:**
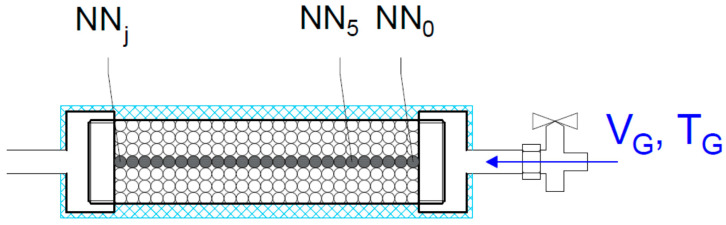
The setup being modeled by the program.

In both models, the first step was to divide a single grain into *N* layers of equal volume. For each of these layers, the radius and surface area were determined. Next, for each layer, an initial temperature (room temperature) was assigned. The operation of the model is shown in Figure 9. The aluminum pipe was divided into NN layers, each with a thickness corresponding to the grain diameter. The simulation used a single grain from each layer, and the air flow rate was reduced proportionally to a single grain. In the next step, the program determined the quantity of gas supplied to the first layer of the bed in unit time. The temperature of the inflowing gas was assumed to be 255 K, this being the lowest temperature recorded during the experiments. Next, the program determined the change in the temperature of the supplied gas resulting from mixing with the room-temperature air surrounding the grain and from the heat received from the grain. Next, the surplus gas at the new temperature surrounding the first grain passed to successive layers of the bed, where it again collected heat. At the next stage, the program computed the quantity of heat supplied to the gas in unit time from the outer layers of the grain. The portion of gas was thus gradually heated until it reached the final layer. The final step of the program enabled the temperature of the gas to be read in a given layer. Model analysis was performed for all four grain classes identified in the outburst at the copper mine.

## 5. Results

### 5.1. Experimental Determination of Specific Heat of the Rock

The experiment to determine the specific heat was carried out 10 times. The results obtained for the specific heat of dolomite are given in Table 2.

The averaged result obtained in the experiments is very close to the values for the specific heat of dolomite reported in the literature, which range from approximately 850 to 950 J/(kg·K) [34,35,36,37,38].

### 5.2. Experimental Determination of Heat Exchange during Gas Flow through the Bed

**Figure 10:**
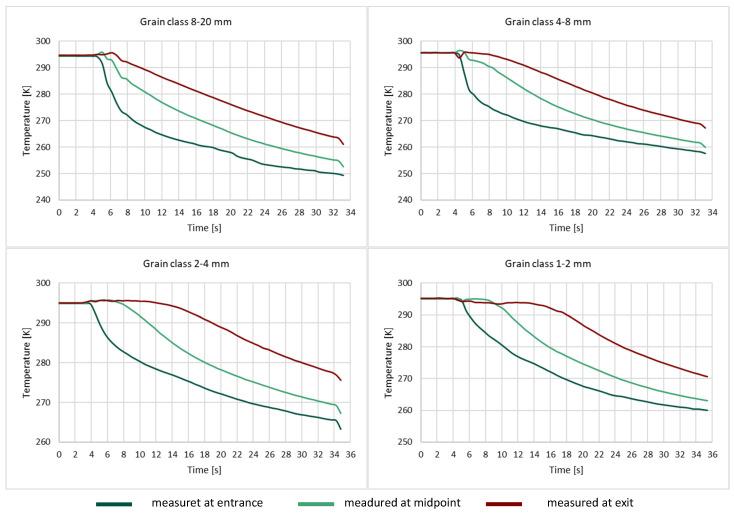
Changes in gas temperature for different grain classes at the entrance to the bed, at the midpoint and at the exit.

The next experiments concerned the flow of gas through a bed enclosed in an aluminum pipe. Temperature graphs for each grain class are given below (Figure 10).

The graphs show how the gas temperature changed over time at three points: at the entrance to the bed, at the midpoint, and at the exit. The temperature was recorded from before the opening of the bottle valve; this is the part of the graph where for approximately 4 s, the temperature at all three measuring points remains stable at 295 K. After the gas bottle was opened, an almost immediate reaction, in the form of a drop in temperature, was observed at the first measuring point at the entrance to the bed. In the central part of the bed, the temperature began to fall after around 0.5–1 s in the case of the 8–20 mm and 4–8 mm grain classes; for the 2–4 mm class, a temperature change was recorded after about 2 s, while for the smallest grain class, the central part of the bed began to cool after about 4 s. The graphs show that in the case of the largest grain class (8–20 mm), the gas temperature at the exit from the bed began to decrease after the shortest time, around 2 s. This means that the gas received relatively little heat from the preceding layers of the bed (successive grains). With decreasing grain size, more time was needed for the final thermometer to begin to record a fall in temperature (4 s for grain class 4–8 mm, 7 s for grain class 2–4 mm, 10 s for grain class 1–2 mm). This trend results from the increasing ratio of the external surface area of the grains to the volume and demonstrates that a bed of smaller grains, with the same apparent volume as the 8–20 mm bed, is able to heat a similar volume of gas much more intensively.

### 5.3. Model Investigation

**Figure 11:**
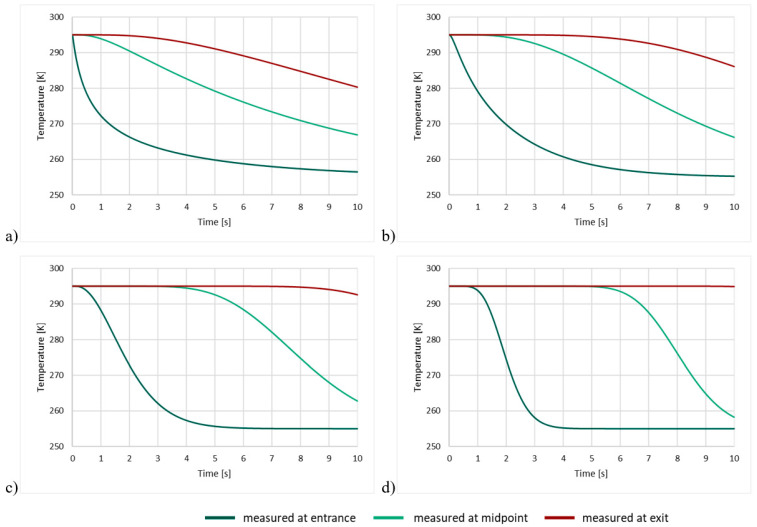
Changes in gas temperature at the entrance to the bed, at the midpoint and at the exit, for grain sizes (**a**) 5 mm, (**b**) 3 mm, (**c**) 1.5 mm, and (**d**) 0.75 mm.

As a result of computer modeling, graphs of temperature at the entrance to the bed, at the midpoint, and at the exit were obtained for conditions corresponding to those of the experiment. Graphs were plotted for grains taken from the middle of the defined size classes: with radii of 5, 3, 1.5, and 0.75 mm. The results are shown in Figure 11 below.

As in the experiment, the change in temperature in the initial modeled layers is almost instantaneous. In the central and end layers, the fall in temperature is delayed, the delay increasing as the grain size decreases.

### 5.4. Comparison of Experiment and Model

**Figure 12:**
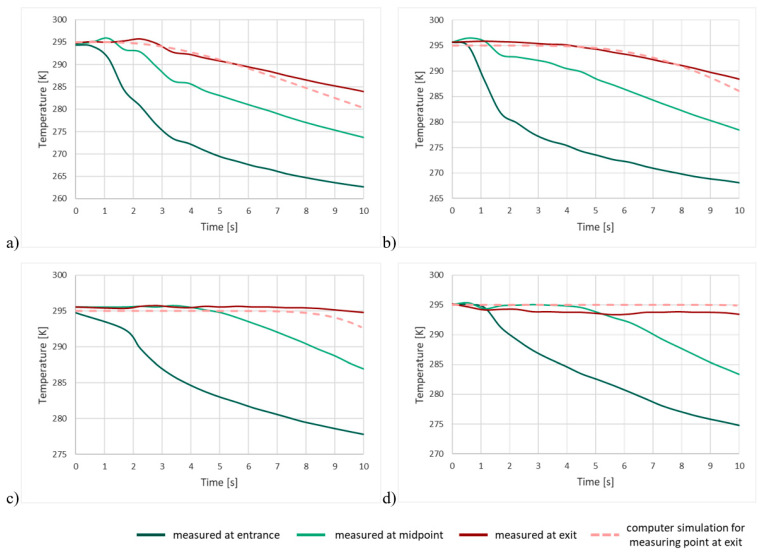
Experimental and simulated changes in gas temperature, for grains of size (**a**) 8–20 mm, (**b**) 4–8 mm, (**c**) 2–4 mm, and (**d**) 1–2 mm.

Both the experimental and model graphs show that significant processes relating to the rate of heat exchange take place in the first 10 s. Therefore, to verify whether the experimental results are in agreement with the simulation, the experimental graphs shown below are overlaid with the simulation graphs for the final measuring point (Figure 12).

**Figure 13:**
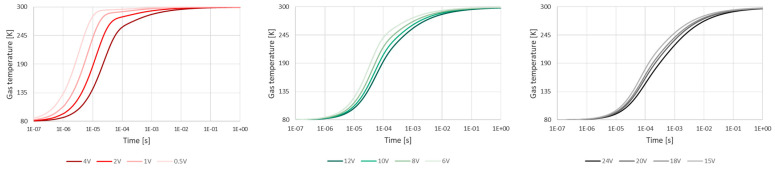
Heat exchange between grains and gas at different values of parameters of the rock–gas system.

When the graphs obtained from the computer model for the final measuring point are overlaid on the experimental graphs, it is observed that the visible change in the gas temperature during the experiment coincides almost perfectly with the modeled exchange of heat between rock and gas. The visible slight differences result mainly from the shape of the grains—in the simulation, they were taken to be ideal spheres arranged in line, while in the experiment, the grains were of random shape and arrangement—and from the size of the grains—in the experiment the grains were of different sizes within a defined interval (from 8 to 20 mm, for example), while in the simulation, a single grain size was assumed (*R* = 5 mm). For the largest grain size class, a visible fall in temperature occurred during the experiment at the final measuring point after approximately 3 s; a visible change in the gas temperature was found in the simulation after the same length of time. For the 4–8 mm grain size class, the falls in temperature in the experiment and in the simulation coincided almost perfectly in the first 10 s. For the 2–4 mm class, the thermometer showed a visible reaction just before the 10th second. In the case of the smallest grain class, the rock heats the gas intensively up to its own temperature for more than 10 s. The agreement between the results of the experiment and those produced by the program confirms the appropriateness of using a spherical model. It is thus possible to use the same model to verify the heat exchange between the grains and the gas that is released from the rock and remains in its vicinity. The modeling of this phenomenon will provide the best picture of the exchange of heat during a rock and gas outburst. The results of the simulation are shown below (Figure 13).

The graphs show that even when the parameters of the rock–gas system are least favorable for gas-induced geodynamic phenomena, when a grain of volume V is surrounded by gas with a volume corresponding to 24 V (porosity 20%, gas pressure 12 MPa), the grain is able to heat the released gas very rapidly (in a time of less than one second). The differences in the time of heating of the gas by a single grain and by the whole bed result from, among other factors, the quantity of gas present during the experiment (approximately 50,000 cm^3^ of cold gas flowed through the entire bed in one second) as well as the nature of contact with the grains: in the case of grain–gas heat exchange, the gas surrounding a grain receives heat only from that grain, whereas in the experiment, a significant quantity of gas comes into contact with successive layers of grains, receiving heat from them and moving onwards at a temperature now higher than the original one; this means that the gas receives smaller and smaller “doses” of heat from successive grains in the layer. Such a rapid exchange of heat between gas and rock implies that the transformation taking place during an outburst—which lasts some tens of seconds—is isothermal in nature.

## 6. Conclusions

This article has described a simplified energy balance for a gas-induced geodynamic phenomenon in compact rocks using the example of an outburst of dolomite and nitrogen. The main source of energy for the outburst is gas at high pressure filling the pores and crevices of the rock [39]. The energy of the gas released during decompression is used to do the work of disintegrating the rock and then transporting it into the mine working space. The decompression of the gas during a geodynamic event is theoretically of an intermediate nature between isothermal and adiabatic. The amount of energy released depends on the actual type of the transformation. Assuming that the parameters of the rock–gas system take the values measured at the site of the outburst (pressure 10 MPa, porosity 20%), the work done by the gas in the case of isothermal transformation is almost three times as high as in the adiabatic case. It is thus necessary to determine the type of thermodynamic transformation so as to establish the parameters of the rock–gas system at which there is a significant danger of a rock and gas outburst. For this reason, an experiment was carried out and computer models were designed to determine how much time was needed to heat the gas to the temperature of the rock. The first step in establishing the nature of the transformation was to determine the specific heat of the studied dolomite, for which a value of 932.74 J/kg∙K was obtained. This result is in agreement with values reported in the literature [34,35,36,37,38]. The fundamental problem addressed in the study was the determination of the rate of heat exchange between the rock and the gas. For this purpose, an experiment was conducted in which cooled gas (at a temperature of around 255 K) flowed at a very high rate (0.05 m^3^/s) through a bed of dolomite at room temperature (295 K). The results of the experiment were compared with those of a computer model implemented in the C++ language. In both the experiment and the computer model, in the case of the dominant grain size class (8–20 mm), a visible drop in temperature at the final measuring point took place after approximately 2–3 s, while for smaller grains, that time was correspondingly longer. The results of the experiment thus agreed with the modeling results to a satisfactory degree. This confirmed the appropriateness of the spherical grain model that was used. The model was additionally used to simulate the exchange of heat between grains and the escaping gas. Based on the simulation, it was determined how much time was needed to heat the gas to a temperature close to that of the rock, assuming the highest recorded parameter values (porosity 20%, gas pressure 12 MPa). This time was found to be slightly under one second. Such rapid heating implies that the transformation taking place during a rock and gas outburst is close to isothermal.

## Figures and Tables

**Table 1:** Volume of gas in terms of the grain volume V.

Gas Volume in Terms of Grain Volume	Example Porosity	Example Pressure
	%	MPa
0.5 V	5	1
1 V	5	2
2 V	10	2
4 V	10	4
6 V	10	6
8 V	20	4
10 V	10	10
12 V	15	8
15 V	15	10
18 V	15	12
20 V	20	10
24 V	20	12

**Table 2:** Experimental values for the specific heat of the rock.

Experiment No.	Specific Heat of Dolomite
	J/kg·K
1	911.25
2	924.23
3	945.01
4	876.41
5	985.74
6	931.38
7	912.34
8	978.29
9	966.89
10	895.88
mean:	932.74
standard deviation:	34.18

## Data Availability

No data were used to support this study.
